# Application of Genetic Algorithm-Based Support Vector Machine in Identification of Gene Expression Signatures for Psoriasis Classification: A Hybrid Model

**DOI:** 10.1155/2021/5520710

**Published:** 2021-09-08

**Authors:** Leili Tapak, Saeid Afshar, Mahlagha Afrasiabi, Mohammad Kazem Ghasemi, Pedram Alirezaei

**Affiliations:** ^1^Department of Biostatistics, School of Public Health, Hamadan University of Medical Sciences, Hamadan, Iran; ^2^Modeling of Noncommunicable Diseases Research Center, Hamadan University of Medical Sciences, Hamadan, Iran; ^3^Research Center for Molecular Medicine, Hamadan University of Medical Sciences, Hamadan, Iran; ^4^Department of Medical Biotechnology, School of Advanced Medical Sciences and Technologies, Hamadan University of Medical Sciences, Hamadan, Iran; ^5^Department of Computer, Hamedan University of Technology, Hamedan, Iran; ^6^Department of Dermatology, Psoriasis Research Center, Hamadan University of Medical Sciences, Hamadan, Iran

## Abstract

**Background:**

Psoriasis is a chronic autoimmune disease impairing significantly the quality of life of the patient. The diagnosis of the disease is done via a visual inspection of the lesional skin by dermatologists. Classification of psoriasis using gene expression is an important issue for the early and effective treatment of the disease. Therefore, gene expression data and selection of suitable gene signatures are effective sources of information.

**Methods:**

We aimed to develop a hybrid classifier for the diagnosis of psoriasis based on two machine learning models of the genetic algorithm and support vector machine (SVM). The method also conducts gene signature selection. A publically available gene expression dataset was used to test the model.

**Results:**

A number of 181 probe sets were selected among the original 54,675 probes using the hybrid model with a prediction accuracy of 100% over the test set. A number of 10 hub genes were identified using the protein-protein interaction network. Nine out of 10 identified genes were found in significant modules.

**Conclusions:**

The results showed that the genetic algorithm improved the SVM classifier performance significantly implying the ability of the proposed model in terms of detecting relevant gene expression signatures as the best features.

## 1. Introduction

Psoriasis is a chronic autoimmune/inflammatory and hyper-proliferative disease with primary manifestations on skin and joints 2]. Psoriasis has been reported to be associated with increased hyperlipidemia, hypertension, coronary artery disease (CAD), diabetes (type II), and obesity [3] as well as the increased risks of stroke and myocardial infarction [4, 5]. Moreover, psoriasis is associated with anxiety, isolation, and mental disorders (e.g., depression), and it reduces socialization for patients and prevents them from having close and intimate relationships [6–8].

Psoriasis can occur at any age, and studies have shown that the inception of the disease takes place between the ages of 20–30 and 50–60 [3]. Nevertheless, most psoriasis patients are <30 years old with a large proportion less than 10 years of age [9], and its prevalence varies according to the climatic/geographical situations such that the Caucasian population has a higher rate and the Asian population has a lower rate among others [10, 11]. There are several phenotypic manifestations including epidermal hyperplasia, angiogenesis, and changed keratinocyte differentiation. Moreover, patients' skin was infiltrated with neutrophils, dendritic cells, and T lymphocytes and chemokine as well as cytokine [12–15]. Visual examination of cutaneous lesion biopsy is the main but inefficient route of diagnosis of psoriasis [14].

While the genetic base of psoriasis has been confirmed, its molecular structure still requires more investigations to be well-enough understood to be useful in the diagnosis of psoriasis [16, 17]. In this regard, quantitative polymerase chain reaction (qPCR) and microarray (high-throughput) techniques have been employed by large-scale studies to reveal the molecular basis of psoriasis and to explore the gene expression patterns in lesional samples compared with nonlesional samples in the disease [15, 18]. In a systematic screening of about 54,000 probe sets conducted by using the Affymetrix HG-U133 Plus 2 platform, a number of 179 unique differential genes (out of 223 probe sets) were identified in the uninvolved psoriatic cutaneous samples and the dysregulated genes were demonstrated to be modulated with the three transcription factors associated with lipid metabolism including sterol regulatory element-binding protein (SREBF), peroxisome proliferator activator receptor alpha (PPARA), and estrogen receptor 2 (ESR2) [16]. Moreover, by screening expressions of approximately 22,000 probe sets using the Affymetrix HG-U133A platform in a study [19], a number of 179 genes among 203 probe sets were detected where the expression of genes in the psoriatic lesion skins changed at least two-fold, and it was shown that there is an association between the Wnt pathway, regulating the stem cell proliferation, and the psoriasis development [15]. Nevertheless, its causative role has remained elusive. There have been detected many up/downregulated probe sets related to lesional samples of skin obtained from psoriasis patients with at least 3-fold changes in expression of some of them like multiple T cell genetic markers and two type I interferon-inducible genes [15, 19]. There are also studies that have compared the level of gene expression of psoriatic and healthy samples in human and mice [20, 21]. While it was observed that the lists of the top 5,000 fold change (up/downregulated probes) were in common with different phenotypes of psoriasis in mice, there is no consistency between different studies in terms of the lists of marker genes. Therefore, developing classification models and identifying target genes are necessary.

During the last decades, machine learning methods have received much attention as they have provided a computer-aided bed for constructing modern classifiers and they have been shown to provide promising results. It has been also shown that the combination of these methods with each other can enhance the prediction power of the classifier. These models can be also used for marker gene selection. Among machine learning methods, support vector machine (SVM) owing to the kernel method has shown promising performance in classification/regression in several medical problems and gene expression studies. SVM fits a hyperplane to separate groups and projects the input space into a higher space by using kernel functions [22]. In this regard, utilizing heuristic approaches like the genetic algorithm (GA) leads to determining a subset of inputs to fit a hyperplane that is most robust among others. GA is an evolutionary algorithm that has received much attention in optimization problems [23]. Mimicking the biology of changes that occur in a DNA sequence like mutation and crossover, GA works by generating an initial set of solutions, which are then assessed through a fitness function. In this way, a subset consisting of the best solutions from the initial solutions is selected and operators like mutation are utilized to generate the consequent sets of solutions [23]. Studies have shown that an automated hybrid system for the diagnosis of diseases like cancer designed based on GA and SVM outperforms the SVM. In this hybrid approach, the GA is used to reduce the dimension of the feature space [24, 25].

To our knowledge, there is no study that proposes hybrid models for the diagnosis of psoriasis. This study proposed a hybrid approach for identifying the psoriasis-associated features (probe sets). The used method is the combination of a GA and SVM for feature selection.

## 2. Methods

### 2.1. Data Source and Preprocessing

A publicly available dataset of psoriasis whole blood transcriptome dataset (available in GEO repository: GSE55201 dataset generated using the Affymetrix U133 Plus (microarray) with platform ID GPL570) was used. This dataset consisted of expression data of 30 healthy controls and 44 psoriasis patients at baseline and 7 psoriasis patients after two weeks of treatment [26]. In this study, the differentially expressed genes (DEGs) between 30 healthy controls and 44 psoriasis patients at baseline samples were determined by using the limma package [27] under R software (version 4.0.3) [28] for subsequent analysis.

### 2.2. Support Vector Machine

The SVM is a machine learning technique that has been developed for classification and regression problems, and it encompasses parts of nonparametric statistics and machine learning. The fundamental idea is to map the covariates in the input space into a space with a higher dimension using some kernel functions, so that a linear regression handles the complex nonlinear regression of the primary input space. The hyperplane equation is **w**.**x** + *b* = 0, and there is a need to minimize the norm of the coefficient vector **w** and to maximize the margin 1/‖**w**‖ between two classes [25].

**Figure 1:**
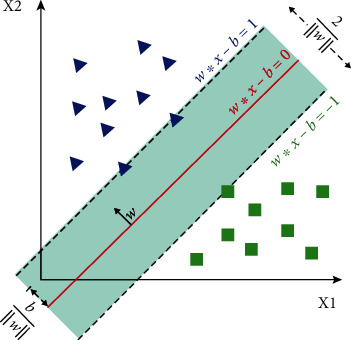
Linear separation of two classes with a support vector machine classifier. Samples on the margin are called the support vectors.

This method utilizes the structural risk minimization principle to fit a hyperplane separating two groups optimally (Figure 1). Let us assume that there is a subject *j* with an input vector of *x*_*j*_ ∈ ℝ^*p*^ with *p* components, which should be classified as psoriasis patient (*y*_*j*_ = −1) or normal subject (*y*_*j*_ = +1). The SVM problem is represented by

(1) $$\begin{array}{c} f\left(x\right)=w\cdot \phi \left(x\right)+b, \end{array}$$

where **w** and *b* stand for the weight vector (regression coefficients) and the bias term, respectively. Then, by considering an *ε*-insensitivity loss function for equation (1), the following optimization problem ((2) and (3)) can be considered a convex optimization problem:

(2) $$\begin{array}{c} \frac{1}{2}w^{T}w+C\sum \xi_{j}+C\sum \xi_{j}^{*}, \end{array}$$

which is optimized given the following restrictions:

(3) $$\begin{array}{c} \begin{array}{c} \left\{\begin{array}{c} w^{T}\phi \left(x_{j}\right)+b-y_{j}\leq \varepsilon +C\xi_{j},\\ y_{j}-w^{T}\phi \left(x_{j}\right)-b\leq \varepsilon +C\xi_{j}^{*}, \end{array}\right.\\ \;j=1,\cdots ,N, \end{array} \end{array}$$

where *ξ*_*j*_^∗^ and *ξ*_*j*_ are nonnegative slack variables (which penalize the training errors in the loss function) with the error tolerance of *ε*. Also, *C* > 0 is the tradeoff parameter which shows the capacity (tuning parameter) and determines the empirical error's degree (lower values of *C* are related to a wider margin and a reduced risk of overfitting an SVM but larger in-sample classification errors). Optimizing of the problem [2] is conducted through minimization of the Lagrange function (4):

(4) $$\begin{array}{c} L\left(w,b,\xi ;\alpha ,\nu \right)=\frac{1}{2}w^{T}w+C\sum \xi_{j}-\sum \alpha_{j}\left\{y_{j}\left(w^{T}\phi \left(x_{j}\right)+b\right)-1+\xi_{j}\right\}-\sum \nu_{j}\xi_{j}, \end{array}$$

with the *α*_*j*_ and *ν*_*j*_ as the Lagrange multipliers. This convex optimization problem can be solved through nonlinear programming tools or via a convex quadratic programming problem in *α*_*j*_. In a nonlinear SVM setting, the score of a subject is computed by substituting the scalar product of the covariates with a kernel function (e.g., polynomial, Gaussian radial basis (GRBF), and exponential radial basis) [29, 30].

### 2.3. Genetic Algorithm

A genetic algorithm is an exploratory algorithm utilized to solve optimization problems. In a GA, a set of candidate solutions (individuals), which is called the initial population, evolved toward better solutions for an optimization problem. Each individual has a set of properties. Solutions are considered strings of 0/1 s (in binary). The solutions are generated using natural evolution, such as inheritance, mutation, and selection. In a GA, chromosomes of a population encode candidate solutions [31].

This evolution is usually created by a random population and occurs in subsequent generations. In each generation, the fitness of each individual in the population is calculated, while several individuals are randomly chosen from the recent population. Then, it is modified to create a new better population. Then, this modification of population is repeated in the next iterations. Usually, the algorithm ends when the maximum number of generations is produced or a predefined level of fitness is obtained for the most recent generated population [23].

In the present study, because we intended to address parameter optimization and feature selection simultaneously, the chromosome (i.e., each individual in the population) was a combination of parameter genes (*C*, *ε*, and other parameters in kernel functions such as *γ* in a kernel function of the form *ϕ*(*x*_*j*_, *x*_*j*′_) = exp(−*γ*‖*x*_*j*_, *x*_*j*′_‖^2^)) and feature gene (*f*_1_, ⋯, *f*_*G*_; *f*_*n*_ ∈ {1, 2, ⋯, *G*} and *G* is the number of candidate features for constructing the model) [32]. Here, a chromosome stands for an individual in GAs, and parameters included in a chromosome are used for SVM modeling [23].

Evaluation and comparison of each candidate were conducted and quantified through a fitness function. In the present study, the accuracy of the SVM classifier of 10-fold cross-validation (CV) was utilized as the fitness function, and greater fitness value was related to a better individual. Given training data ((*x*_*j*_, *y*_*j*_) ∈ (ℝ^*p*^, {−1, +1}) for *N* subjects), the objective function can be calculated by equation (4). The accuracy of 10-fold CV for the SVM was calculated by

(5) $$\begin{array}{c} \text{Accuracy}=\frac{\text{True positive}+\text{True negative}}{\text{total sample}}, \end{array}$$

which is the proportion of correct predictions among the total number of samples examined (true positive indicates the number of psoriasis patients who were predicted as patients truly, and true negative indicates the number of healthy cases who were predicted as healthy truly).

### 2.4. Hybrid GA-SVM Model and Tuning Parameters

**Figure 2:**
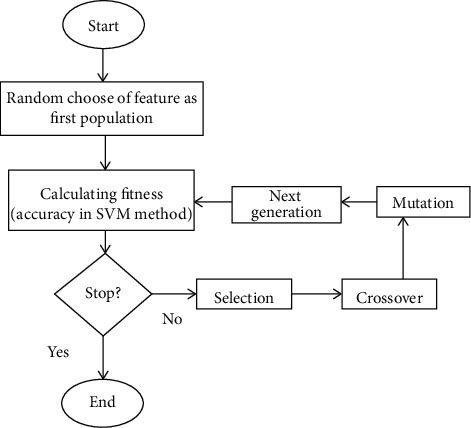
Overview of the hybrid GA-SVM model.

This study utilized a method for enhancing the performance of the SVM. The enhancement is to select the best subset of features and to optimize the parameters of the model. GA was utilized to handle both aforementioned aspects in the SVM classification problem simultaneously for psoriasis diagnosis. In GA, the fitness function was the accuracy of the SVM classifier. The GA algorithm was used to optimize the objective function defined for the SVM classifier (equation (4)) and to find suitable features (feature selection) for the diagnosis of psoriasis and detecting a healthy/patient person.  Figure 2 shows the block diagram of the hybrid GA-SVM model.

In the GA used, the number of populations was five. The solutions were represented in binary as strings of 0 s and 1 s. The value 0 indicated that the feature/attribute was not selected and value 1 indicated that the feature/attribute was selected. The roulette wheel model, single-point crossover operator with 0.8 of crossover rate, and mutation operator with 0.01 of the mutation rate were used for the selection of the appropriate chromosomes to produce the next generation.

In the roulette wheel selection, the probability of selecting an individual upbringing of the next generation is proportional to its fitness. A better fitness (here, greater prediction accuracy) is related to a higher probability of selecting an individual. Selecting a solution/individual can be considered spinning roulette with pockets for each individual with sizes depending on their probability (*p*_*j*_ = *f*_*j*_(the fitness of *j*th individual)/∑_*j*=1_^*G*^*f*_*j*_, where *G* is the size of the current generation).

To optimize the parameters of the SVM, they were encoded with binary chains on two fix search intervals of

(6) $$\begin{array}{c} \begin{array}{c} C_{\max}<C<C_{\min},\\ \gamma_{\max}<\gamma <\gamma_{\min}. \end{array} \end{array}$$

Thus, a 32-bit encoding scheme of *C* (i.e., *C*_*b*1_, ⋯, *C*_*b*32_) and *γ* where

(7) $$\begin{array}{c} C_{b}=\overset{32}{\underset{i=1}{\sum }}C_{bi}2^{i-1}=\frac{g_{\max}\left(C-C_{\min}\right)}{C_{\max}-C_{\min}},\\ \gamma_{b}=\overset{32}{\underset{i=1}{\sum }}\gamma_{bi}2^{i-1}=\frac{g_{\max}\left(\gamma -\gamma_{\min}\right)}{\left(\gamma_{\max}-\gamma_{\min}\right)}, \end{array}$$

with *g*_max_ = 2^32^ − 1 was considered [33].

### 2.5. Evaluation Criteria

We exerted the method on 74 samples (50 samples for training and 24 samples for testing).

In this study, the total accuracy and the area under the ROC curve (AUC) were used to evaluate the performance of the models.

(8) $$\begin{array}{c} \text{Accuracy}=\frac{\text{True positive}+\text{True negative}}{\text{total sample}}. \end{array}$$

Analyses were performed by using the MATLAB software programming [34].

### 2.6. Protein-Protein Interaction (PPI) Network

The protein-protein interaction (PPI) network was constructed using the STRING version 11.0 [35] with a confidence cutoff of 0.7 for selected probes with GA+SVM. The constructed PPI network was visualized and analyzed using Cytoscape version 3.6.0 [36]. The CytoHubba plugin under Cytoscape software was used to determine the hub genes. Modules of the constructed network were evaluated with the MCODE plugin under Cytoscape software with cutoff criteria of the number of nodes more than 5 and MCODE score more than 3 and default parameters (node score cutoff = 0.2, *K*‐core = 2, degree cutoff = 2, and max depth = 100).

### 2.7. Kyoto Encyclopedia of Genes and Genomes (KEGG) Pathway and Gene Ontology (GO) Enrichment Analysis

The KEGG pathway and GO enrichment analysis including the biological process, molecular function, and cellular component were performed using the DAVID database (https://david.ncifcrf.gov/) for selected probes with GA+SVM.

## 3. Results

### 3.1. Comparison of the Models

**Figure 3:**
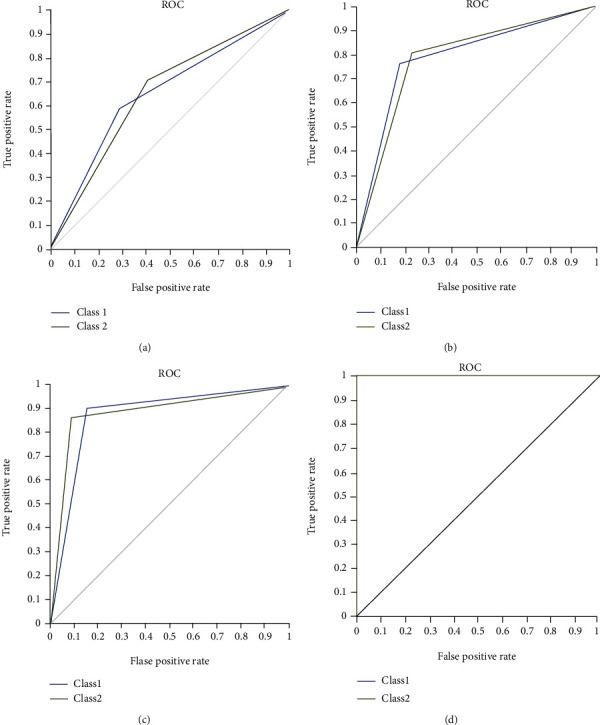
The ROC curves of the four scenarios of classification of psoriasis patients using (a) SVM with 54,657 features, (b) GA+SVM with 27,265 features, (c) SVM with 445 features, and (d) GA+SVM with 181 features.

Table 1 shows the performance of the SVM and GA-SVM. The first two lines of the table are related to the original 54,675 probe sets which led to a total accuracy and AUC of 62.500% and 0.625, respectively. By using features selected by GA (27,265 features), the total accuracy and AUC of the SVM increased to 79.167% and 0.792, respectively. Moreover, after an initial screening of the probe sets using the limma package based on the ∣FC | ≥1 and adjusted *P* value < 0.05, a number of 445 DEGs were selected for further evaluation. Then, the SVM and GA-SVM were trained using them. According to the results (Table 1), the total accuracy and AUC of the SVM using 445 DEGs were 87.500% and 0.878, respectively. After feature selection using GA, the number of 181 probe sets was selected and the total accuracy and AUC of the SVM (using 181 features) increased to 100 and 1, respectively. Also, ROC curves related to the four scenarios are provided in Figure 3.

### 3.2. PPI Network Analysis

**Figure 4:**
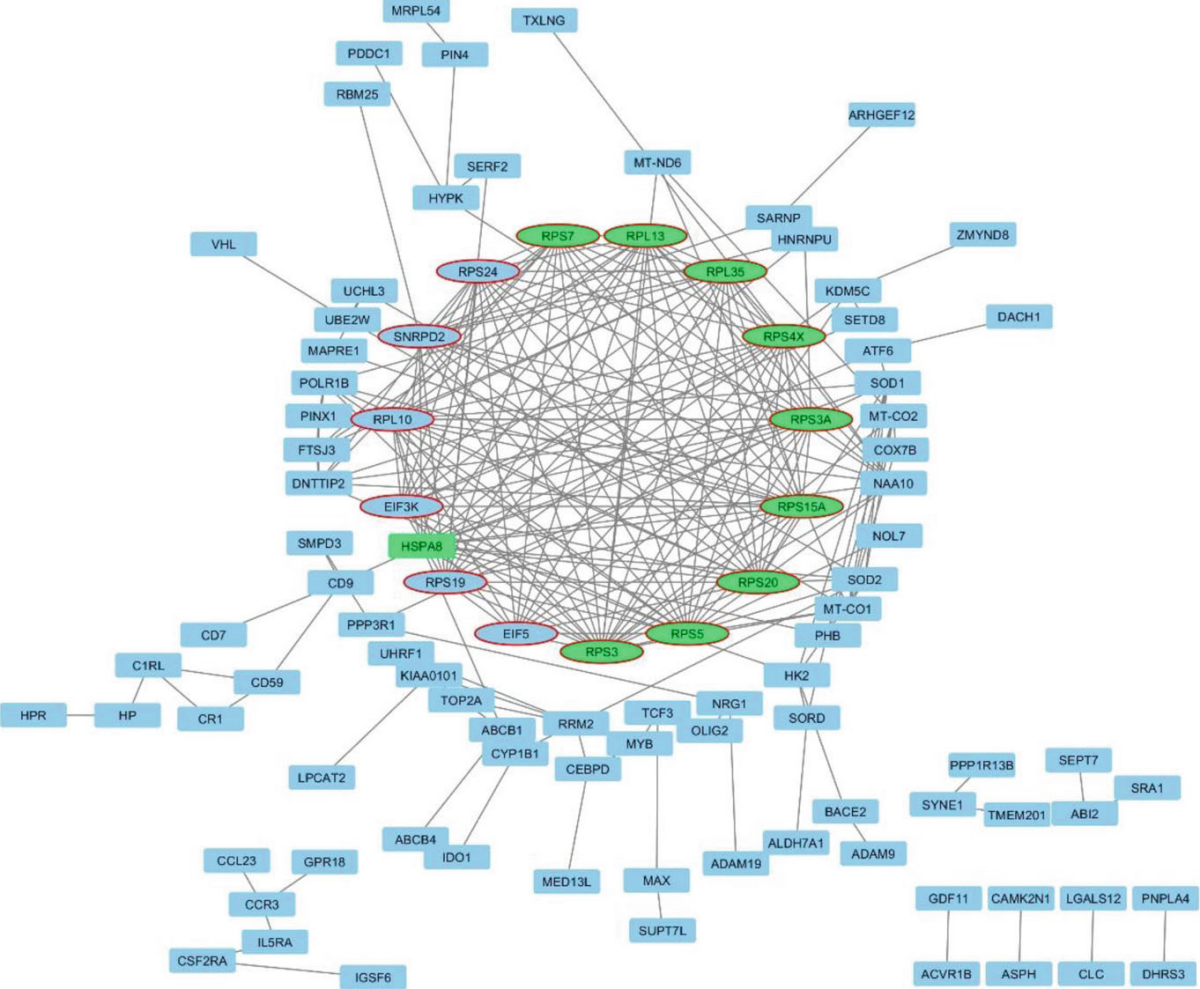
Protein-protein interaction network. The PPI network was constructed using STRING and visualized with Cytoscape. The selected top 10 genes with a high degree were shown in green. The nodes related to the significant module were shown in ellipse shapes.

The constructed PPI network for selected probes with GA+SVM, consisting of 100 nodes and 244 edges, is shown in Figure 4. The top 10 genes with a high degree including ribosomal protein S3 (RPS3), ribosomal protein S5 (*RPS5*), ribosomal protein S20 (RPS20), ribosomal protein S15a (RPS15A), ribosomal protein S3A (RPS3A), X-linked ribosomal protein S4 (RPS4X), ribosomal small subunit protein 7 (RPS7), ribosomal protein L13 (RPL13), ribosomal protein L35 (RPL35), and heat shock protein family A member 8 (HSPA8) were selected.

Evaluation of the PPI network indicated that a significant module with a score of 14.570 consisted of 15 nodes and 102 edges. As seen in Figure 4, among the top 10 genes, 9 hub genes including RPS3, RPS5, RPS20, RPS15A, RPS3A, RPS4X, RPS7, RPL13, and RPL35 were found in the significant module.

### 3.3. GO and KEGG Pathway Analysis

**Figure 5:**
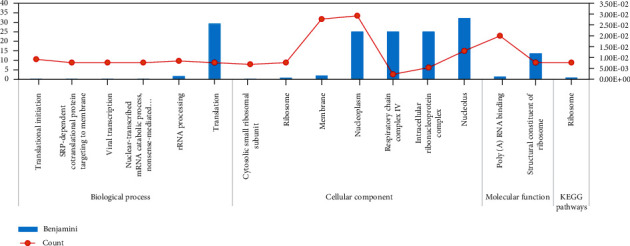
Gene Ontology and KEGG pathway enrichment analysis. The KEGG pathway and GO enrichment analysis for selected probes with GA+SVM were performed using the DAVID database.

**Figure 6:**
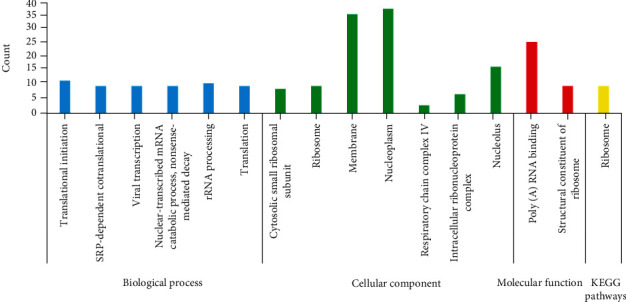
Gene Ontology and KEGG pathway enrichment analysis.

**Figure 7:**
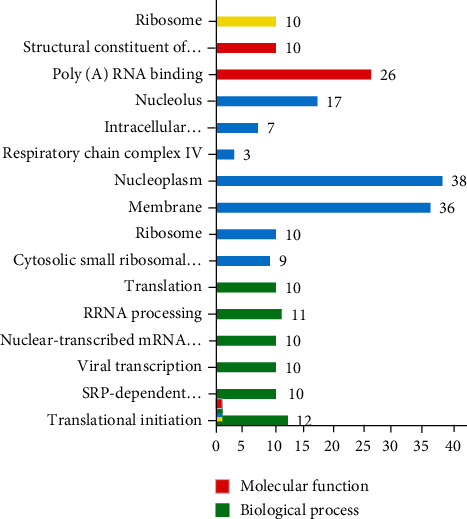
Molecular *g* = function and biological process.

The KEGG pathway analysis (Figures 5–7) indicated that the ribosome pathway was significantly enriched in 181 selected genes. GO enrichment analysis showed that the structural constituent of the ribosome and poly(A) RNA binding were enriched in molecular function GO terms; cytosolic small ribosomal subunit, ribosome, membrane, nucleoplasm, respiratory chain complex IV, intracellular ribonucleoprotein complex, nucleolus, and small ribosomal subunit were enriched in cellular compartments; and translational initiation, SRP-dependent cotranslational protein targeting to membrane, viral transcription, nuclear-transcribed mRNA catabolic process, nonsense-mediated decay, rRNA processing, and translation were enriched in biological process terms.

## 4. Discussions

This study proposed a hybrid machine learning model to provide a precise psoriasis prediction model, using the gene expression profiles of human samples. The results of the present study showed the ability of the proposed model to discriminate psoriasis cases from normal controls. To evaluate the performance of the model, the predicted diagnosis (binary predicted response: psoriasis vs. control) returned by the proposed model during the validation stage (i.e., over test set) was compared against the true target value (i.e., known diagnosis of binary observed response: psoriasis vs. control). The best prediction model would return a high AUC and high prediction accuracy. The traditional SVM (without GA) was also trained and compared with the hybrid model. The finding of this study showed a higher performance for the proposed hybrid prediction model and showed that GA has significantly improved the performance of the SVM classifier by achieving a total accuracy of 100%.

By comparing the results of the proposed GA+SVM of this study with those of Le et al. [37] who compared the performance of four classifiers of the random forest, naïve Bayes, *K*-nearest neighborhood, and SVM for the classification of psoriasis and achieved a 98.3% total accuracy for the random forest classifier (using all 54,675 probe sets and a larger sample size of *n* = 180 compared with the present study with *n* = 74), a greater accuracy was obtained based on 181 probe sets using a much smaller sample size (*n* = 74) indicating the need for noteworthy performance of the hybrid models. Moreover, feature selection based on the genetic algorithm reduces the dimension of the feature space, so the redundant, noisy, or irrelevant data are removed, the quality of the data and the accuracy of the resulting model improve due to searching from a population of points instead of a single point, and finally the selected feature set avoids wasting of resources in the next round of information collection or throughout utilization [38]. Moreover, in other studies, GA+SVM has been shown to outperform the traditional SVM in detecting other diseases or other outcomes [22, 24]. So it is suggested to use and evaluate the performance of other hybrid models. Apparently, the proposed technique illustrates competitive performance against the state-of-the-art models.

PPI network analysis identified a set of 9 hub genes (including RPS3, RPS5, RPS20, RPS15A, RPS3A, RPS4X, RPS7, RPL13, and RPL35) that is proposed to be associated with psoriasis or to be differentially expressed in psoriasis samples. The protein encoded by RPS3 has an important role in DNA repair through the cleavage of damaged DNA. Moreover, RPS3 enhances the inflammatory response through proteasomal degradation of I*κ*B*α* [39, 40].

RPS5 encodes the nucleotide-binding protein which is activated proteolytically with PBS1. RPS5 regulates several biological processes such as proliferation and differentiation. Furthermore, RPS5 suppresses the inflammatory responses induced by lipopolysaccharide [41, 42]. The protein encoded by RPS20 plays a role in P53 activation through the Mdm2 binding and ribosomal biogenesis [43, 44]. RPS7 regulates the cell cycle and apoptosis via the MDM2-P53 interaction and through the regulation of MAPK and PI3K/AKT signaling pathways [45]. The protein encoded by PS4X is one of the components of the ribosomal small subunit complex. PS4X plays an essential role in biological processes such as proliferation and translation [46, 47]. The protein encoded by RPL13 plays an essential role in pre-rRNA processing and ribosome assembly [48]. RPL35 as an important component of the ribosomal large subunit plays an essential role in protein translation [49]. RPS15A has an essential role in mRNA binding to the small subunit of the ribosome and control of the cell cycle [50]. RPS15A activates the NF-*κ*B signaling pathway by inducing I*κ*B-*α* degradation [51]. RPS3A has an essential role in the regulation of translation, apoptosis, and differentiation. RPS3A also plays a role in NF-*κ*B signaling pathway enhancement [52, 53]. In this study, KEGG pathway analysis indicated that the ribosome pathway was significantly enriched for selected genes. The structural constituent of the ribosome and poly(A) RNA binding were significantly enriched in molecular function GO terms. Translational initiation, SRP-dependent cotranslational protein targeting to membrane, nonsense-mediated decay, viral transcription, rRNA processing, translation, and nuclear-transcribed mRNA catabolic process were enriched in biological process GO terms.

A similar study showed that the ribosome pathway might be associated with inflammation [54]. Moreover, results of Li et al.'s study indicated that selected DEGs in psoriasis were enriched in the ribosomal pathway [55]. rRNA processing is required for 28S and 5.8S rRNA maturation and proper ribosome biogenesis and plays a role in innate immune signaling [56, 57]. Wu et al. in their study showed that in ankylosing spondylitis as an autoimmune disease, the structural constituent of the ribosome, SRP-dependent cotranslational protein targeting to membrane, nonsense-mediated decay, translation, viral transcription, nuclear-transcribed mRNA catabolic process, poly(A) RNA binding, and translational initiation term were enriched [58].

## 5. Conclusions

This study proposed a hybrid method of GA and SVM for the diagnosis of psoriasis. The proposed method was assessed using a real dataset and compared with the conventional SVM. The current study results revealed that the hybrid method outperformed the traditional SVM demonstrating the feasibility of identifying the best features using GA. Finally by considering the results of such hybrid method, assessing other heuristic approaches such as ant colony or particle swarm optimization is suggested for future studies.

## Figures and Tables

**Table 1:** The performance criteria of the SVM and GA-SVM.

Number of used features	Method	Total accuracy (%)	AUC^∗^
54,657	SVM	62.500	0.625
27,265	GA+SVM	79.167	0.792
445	SVM	87.500	0.878
181	GA+SVM	100.000	1.000

^∗^Area under the ROC curve.

## Data Availability

A publicly available dataset was analyzed in this study. This data can be found at https://www.ncbi.nlm.nih.gov/geo/ (the NCBI Gene Expression Omnibus).
